# Impact of *YTHDF1* gene polymorphisms on Wilms tumor susceptibility: A five‐center case‐control study

**DOI:** 10.1002/jcla.23875

**Published:** 2021-06-21

**Authors:** Yanfei Liu, Huiran Lin, Rui‐Xi Hua, Jiao Zhang, Jiwen Cheng, Suhong Li, Haixia Zhou, Zhenjian Zhuo, Jun Bian

**Affiliations:** ^1^ Department of Pathology Xi'an Children’s Hospital, The Affiliated Children’s Hospital of Xi'an Jiaotong University Xi'an China; ^2^ Faculty of Medicine Macau University of Science and Technology Macau China; ^3^ Department of Pediatric Surgery Guangzhou Institute of Pediatrics Guangdong Provincial Key Laboratory of Research in Structural Birth Defect Disease Guangzhou Women and Children’s Medical Center Guangzhou China; ^4^ Department of Pediatric Surgery The First Affiliated Hospital of Zhengzhou University Zhengzhou Henan China; ^5^ Department of Pediatric Surgery The Second Affiliated Hospital of Xi'an Jiaotong University Xi'an China; ^6^ Department of Pathology Children Hospital and Women Health Center of Shanxi Taiyuan China; ^7^ Department of Hematology The Second Affiliated Hospital and Yuying Children’s Hospital of Wenzhou Medical University Wenzhou China; ^8^ Department of General Surgery Xi'an Children’s Hospital, Xi'an Jiaotong University Affiliated Children’s Hospital Xi'an China

**Keywords:** case‐control study, polymorphism, risk, Wilms tumor, *YTHDF1*

## Abstract

**Background:**

Wilms tumor is the most frequent renal malignancy in children. YTHDF1 is associated with the development of several kinds of cancers, yet whether common variants of the *YTHDF1* gene influence Wilms tumor risk is unknown. We present, here, a hospital‐based case‐control study specifically designed to investigate the role of *YTHDF1* genetic variants on Wilms tumor.

**Methods:**

We successfully genotyped samples of 408 Wilms tumor cases and 1198 controls which were collected from five hospitals across China. The unconditional logistic regression was adopted to analyze the contributions of *YTHDF1* gene single nucleotide polymorphisms (SNPs) to the risk of Wilms tumor. The odds ratio (OR) and 95% confidence interval (CI) were generated to evaluate the conferring risk of *YTHDF1* gene SNPs (rs6011668 C>T, rs6090311 A>G).

**Results:**

Neither of the two SNPs could contribute to the risk of Wilms tumor. A negative association was also detected in the combined effects of protective genotypes on Wilms tumor risk. The stratification analysis revealed that compared with those with CC genotype, rs6011668 CT/TT genotype was associated with increased Wilms tumor risk in those ≤18 months (OR = 1.54, 95% CI = 1.02–2.30, *p* = 0.038), and with decreased Wilms tumor risk in those >18 months (OR = 0.70, 95% CI = 0.50–0.97, *p* = 0.034).

**Conclusion:**

Our present work sheds some light on the potential role of *YTHDF1* gene polymorphisms on Wilms tumor risk.

## Graphical abstract

*YTHDF1* gene SNPs (rs6011668 C>T, rs6090311 A>G) could not contribute to the risk of Wilms tumor. A negative association was also detected in the combined effects of protective genotypes on Wilms tumor risk. The stratification analysis revealed that compared with those with CC genotype, rs6011668 CT/TT genotype was associated with increased Wilms tumor risk in those ≤18 months, and with decreased Wilms tumor risk in those >18 months.

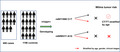

## 1 INTRODUCTION

Wilms tumor (nephroblastoma) is a solid kidney tumor that mainly affects children.^1^ It is a tumor of embryonic origin that originates from abnormal differentiation in primitive cells during fetal nephrogenesis.^2^ The incidence of Wilms tumor is higher in the United States compared to that of Wilms tumor in China, there being about 7 new cases per million children compared to 3.3 per million.^3^, ^4^ Nearly 80% of cases are diagnosed before the age of five.^5^ With the advancement of medical treatment, the 5‐year survival of favorable histology Wilms tumor has exceeded over 90%.^6^ For patients with unfavorable Wilms tumors, the prognosis is still disappointing.^6^

Wilms tumor is a complex disease characterized by the broad heterogeneity of molecular genetic alterations.^7^, ^8^ The *WT1* gene was discovered as the first identified Wilms tumor mutated gene in 1990.^9^ Subsequently, mutations in the genes *CTNNB1*, *AMER1*, and *DROSHA* were demonstrated as Wilms tumor risk factors.^7^, ^10^, ^11^, ^12^ Genome‐wide analysis, as well as case‐control studies also identified additional Wilms tumor risk loci.^13^, ^14^, ^15^, ^16^ However, all the identified gene mutations only explain a small portion of Wilms tumor origin. Characterization of more variants will further clarify the etiology of Wilms tumor.

N6‐methyladenosine (m^6^A) is one of the most seen internal modifications in mRNAs.^17^, ^18^, ^19^, ^20^ The m^6^A is a dynamic and reversible process where three groups of proteins take part in, including methyltransferases, demethylases, and m^6^A‐specific binding proteins.^21^ The binding proteins mainly include YTH‐family proteins YTHDF1‐3, YTHDC1‐2, eukaryotic initiation factor eIF3, and insulin‐like growth factor 2 mRNA‐binding proteins IGF2BP1‐3.^22^ In the cytosol, YTHDF1 interacts with translation initiation factors eIF3 and eIF4A3 to promote the translation process of m^6^A‐modified mRNAs.^23^ YTHDF1 has been implicated in several types of cancers.^24^, ^25^, ^26^ However, whether *YTHDF1* gene variants are related to the risk of Wilms tumor is not reported yet. The objective of our case‐control study was to determine whether the *YTHDF1* gene variants are associated with Wilms tumor risk.

## 2 METHODS

### 2.1 Sample selection

The study was approved by the Ethics Committee of Guangzhou Women and Children's Medical Center. We carried out the entire work in accordance with the ethical guidelines of the tenets of the Declaration of Helsinki. The cases all had newly diagnosed, histologically confirmed, and previously untreated Wilms tumor. Controls were randomly selected from hospital visitors who were living in the same area and were free of Wilms tumor when being enrolled. All study participants’ guardians provided written informed consent. A total of 414 cases diagnosed with Wilms tumor and 1199 hospital‐based controls were included (Table S1). They were recruited from five hospitals (Guangzhou Women and Children's Medical Center, The Second Affiliated Hospital and Yuying Children's Hospital of Wenzhou Medical University, The First Affiliated Hospital of Zhengzhou University, Second Affiliated Hospital of Xi'an Jiao Tong University, and Shanxi Provincial Children's Hospital) in five different cities of China. Detailed information regarding sample selection was accessible in our previous studies.^27^, ^28^

### 2.2 Polymorphism selection and genotyping

We chose two SNPs of *YTHDF1* gene, rs6011668 C>T and rs6090311 A>G, to genotype. The reasons for choosing these two SNPs were described in our previous study.^29^ To be specific, the following criteria were adopted to choose potentially functional polymorphisms: (1) the minor allele frequency (MAF) reported in HapMap was >5% for Chinese Han subjects; (2) putative functional potentials SNPs located in the 5’‐ flanking region, exon, 5’‐ untranslated region (5’ UTR), and 3’ UTR, which might affect transcription activity or binding capacity of the microRNA binding site; (3) SNPs in low linkage disequilibrium with each other (*R* ^2^ < 0.8). Both the two SNPs (rs6011668 C>T and rs6090311 A>G) are located in the transcription factor binding sites (TFBS). There is no significant linkage disequilibrium (LD) (*R* ^2^ < 0.8) between rs6011668 C>T and rs6090311 A>G in *YTHDF1* gene (*R* ^2^ = 0.094). Genomic DNA was isolated from peripheral blood according to the standard protocol. Genotyping was performed by TaqMan SNP Genotyping Assay, by means of an ABI 7900HT (Applied Biosystems).^30^ In each genotyping plate, we inserted negative control samples (water) to ensure the quality of genotyping. 10% of randomly selected replicates from the study sample were re‐genotyped. Concordance rates for the original and replicate samples were 100%.

### 2.3 Statistical analysis

For the analyzed SNPs, a goodness‐of‐fit χ^2^ test was used to test for deviations from Hardy‐Weinberg equilibrium (HWE). To test the difference in the distribution of demographic variables between cases and controls, a Chi‐square test for categorical variables and a Student *t*‐test for continuous variables were conducted. The association between the SNPs and Wilms tumor risk was determined using unconditional logistic regression computing odds ratios (ORs) and 95% confidence intervals (CIs). Stratified analyses were carried out across the strata of the following factors: age, sex, and clinical stages. In all analyses, a two‐tailed *p* value <0.05 was considered statistically significant. Statistical calculations were done with the SAS statistical software package version 9.1 (SAS Institute Inc.).^31^

## 3 RESULTS

### 3.1 Effect of *YTHDF1* gene SNPs on Wilms tumor risk

**Table ****S1** gives information on the baseline characteristics of the included cases and controls. Similar distributions of age (*p* = 0.118) and gender (*p* = 0.218) were observed between cases and controls. Of all the included samples (414 cases and 1199 controls), we successfully genotyped 408 Wilms tumor cases and 1198 controls. The genotype distribution of rs6011668 C>T and rs6090311 A>G polymorphisms and their relationship with Wilms tumor risk are listed in Table 1. As expected, the genotype distributions of rs6011668 C>T (*P* for HWE = 0.490) and rs6090311 A>G (*P* for HWE = 0.378) polymorphism in controls did not deviate from HWE. We evaluated the association of rs6011668 C>T and rs6090311 A>G with Wilms tumor risk but did not find statistical significance in all genotype models. Non‐significant association results remained unchanged after adjusting by age and sex. We then allocated rs6011668 CT/TT and rs6090311 AG/GG genotypes as protective genotypes. Compared to 0 protective genotype, 1, 2, and 1–2 protective genotypes could not decrease Wilms tumor risk.

**TABLE 1:** Association between *YTHDF1* gene polymorphisms and Wilms tumor susceptibility

Genotype	Cases (*N* = 408)	Controls (*N* = 1198)	*p* ^a^	Crude OR (95% CI)	*P*	Adjusted OR (95% CI)^b^	*p* ^b^
rs6011668 C>T (HWE = 0.490)							
CC	300 (73.53)	868 (72.45)		1.00		1.00	
CT	104 (25.49)	307 (25.63)		0.98 (0.76–1.27)	0.879	0.97 (0.75–1.26)	0.826
TT	4 (0.98)	23 (1.92)		0.50 (0.17–1.47)	0.208	0.49 (0.17–1.42)	0.189
Additive			0.473	0.92 (0.73–1.16)	0.473	0.91 (0.72–1.15)	0.422
Dominant	108 (26.47)	330 (27.55)	0.674	0.95 (0.74–1.22)	0.674	0.94 (0.73–1.21)	0.619
Recessive	404 (99.02)	1175 (98.08)	0.202	0.51 (0.17–1.47)	0.211	0.49 (0.17–1.43)	0.193
rs6090311 A>G (HWE = 0.378)							
AA	162 (39.71)	458 (38.23)		1.00		1.00	
AG	188 (46.08)	577 (48.16)		0.92 (0.72–1.18)	0.508	0.92 (0.72–1.18)	0.516
GG	58 (14.22)	163 (13.61)		1.01 (0.71–1.43)	0.973	1.02 (0.72–1.45)	0.911
Additive			0.767	0.98 (0.83–1.16)	0.824	0.99 (0.84–1.17)	0.875
Dominant	246 (60.29)	740 (61.77)	0.597	0.94 (0.75–1.18)	0.597	0.94 (0.75–1.19)	0.623
Recessive	350 (85.78)	1035 (86.39)	0.758	1.05 (0.76–1.45)	0.758	1.07 (0.77–1.47)	0.698
Protective genotypes^c^							
0	98 (24.02)	262 (21.87)		1.00		1.00	
1	266 (65.20)	802 (66.94)		0.89 (0.68–1.16)	0.383	0.89 (0.68–1.16)	0.376
2	44 (10.78)	134 (11.19)	0.433	0.88 (0.58–1.33)	0.536	0.87 (0.58–1.32)	0.516
0	98 (24.02)	262 (21.87)		1.00		1.00	
1–2	310 (75.98)	936 (78.13)	0.369	0.89 (0.68–1.15)	0.369	0.88 (0.68–1.15)	0.359

Abbreviations: OR, odds ratio; CI, confidence interval; HWE, Hardy‐Weinberg equilibrium.

^a^ χ^2^ test for genotype distributions between Wilms tumor patients and controls.

^b^ Adjusted for age and sex.

^c^ Protective genotypes were carriers with rs6011668 CT/TT and rs6090311 AG/GG genotypes.

### 3.2 Stratification analysis

We next determined the association between *YTHDF1* gene polymorphisms and susceptibility to Wilms tumor in subgroups separated by age, sex, and clinical stages (Table 2). For rs6011668, the CT/TT genotype was associated with increased Wilms tumor risk in those ≤18 months (OR = 1.54, 95% CI = 1.02–2.30, *p* = 0.038), or with decreased Wilms tumor risk in those >18 months (OR = 0.70, 95% CI = 0.50–0.97, *p* = 0.034), in comparison to CC genotype. No significant associations were found between rs6090311 AG/GG or 1–2 protective genotypes and the risk of Wilms tumor, in all subgroups.

**TABLE 2:** Stratification analysis for the association between *YTHDF1* gene polymorphisms and Wilms tumor susceptibility

Variables	rs6011668 (cases/controls)		AOR (95% CI)^a^	*p* ^a^	rs6090311 (cases/controls)		AOR (95% CI)^a^	*p* ^a^	Protective genotypes (cases/controls)		AOR (95% CI)^a^	*p* ^a^
	CC	CT/TT			AA	AG/GG			0	1–2		
Age, month												
≤18	91/343	49/122	1.54 (1.02–2.30)	0.038	48/178	92/287	1.19 (0.80–1.78)	0.381	24/100	116/365	1.34 (0.82–2.20)	0.243
>18	209/525	59/208	0.70 (0.50–0.97)	0.034	114/280	15/453	0.85 (0.64–1.13)	0.274	74/162	194/571	0.75 (0.55–1.04)	0.082
Sex												
Females	140/363	52/158	0.85 (0.59–1.24)	0.401	80/207	112/314	0.92 (0.66–1.29)	0.642	47/114	145/407	0.86 (0.59–1.28)	0.463
Males	160/505	56/172	1.02 (0.72–1.45)	0.896	82/251	134/426	0.97 (0.71–1.33)	0.838	51/148	165/529	0.90 (0.63–1.30)	0.588
Clinical stages												
I	92/868	45/330	1.28 (0.87–1.87)	0.209	58/458	79/740	0.85 (0.59–1.22)	0.373	27/262	110/936	1.14 (0.73–1.77)	0.572
II	87/868	27/330	0.81 (0.51–1.27)	0.350	43/458	71/740	1.03 (0.69–1.53)	0.877	29/262	85/936	0.82 (0.53–1.28)	0.387
III	73/868	21/330	0.76 (0.46–1.25)	0.279	39/458	55/740	0.87 (0.57–1.33)	0.513	27/262	67/936	0.70 (0.44–1.12)	0.133
IV	35/868	12/330	0.89 (0.46–1.75)	0.744	18/458	29/740	1.00 (0.55–1.82)	0.993	11/262	36/936	0.92 (0.46–1.82)	0.802
I+II	179/868	72/330	1.05 (0.77–1.41)	0.777	101/458	150/740	0.92 (0.70–1.22)	0.576	56/262	195/936	0.97 (0.70–1.35)	0.857
III+IV	108/868	33/330	0.81 (0.53–1.21)	0.300	57/458	84/740	0.91 (0.64–1.30)	0.597	38/262	103/936	0.76 (0.51–1.14)	0.182

Abbreviations: AOR, adjusted odds ratio; CI, confidence interval.

^a^ Adjusted for age and sex, omitting the corresponding stratify factor.

## 4 DISCUSSION

Emerging epidemiological evidence has shown the implication of genetic variants in Wilms tumor risk. To fully unearth the genetic spectrum of Wilms tumor still is a challenge that remains. The current work provided a collection of evidence regarding the role of *YTHDF1* gene polymorphisms on risk of Wilms tumor.

*YTHDF1* gene resides in chromosome 20q11. Prior studies have found YTHDF1 to be associated with cancer. The up regulation of YTHDF1 is detected in ovarian cancer and associated with adverse prognosis. YTHDF1 facilitates tumorigenesis and metastasis of ovarian cancer via augmenting the translation of EIF3C.^32^ YTHDF1 is frequently amplified in hepatocellular carcinoma (HCC) tissues and significantly associated with the prognosis of HCC patients. Mechanism analysis revealed that YTHDF1 can accelerate the translational output of FZD5 mRNA in an m^6^A‐dependent manner and function as an oncogene through the WNT/β‐catenin pathway.^33^ METTL3 facilitates oral squamous cell carcinoma tumorigenesis by strengthening the c‐Myc stability via YTHDF1‐mediated m^6^A modification.^34^ Shi et al.^35^ demonstrated that deficiency of YTHDF1 inhibited non‐small cell lung cancer cell proliferation and xenograft tumor formation. Unexpectedly, they observed that the high expression of YTHDF1 was related to better clinical outcomes. Nishizawa et al.^36^ found that high YTHDF1 expression was linked to a significantly more reduced overall survival rate in colorectal cancer patients. Molecular mechanism experiments revealed that c‐Myc could drive YTHDF1 to facilitate cancer proliferation.

Epidemiology reports of *YTHDF1* gene SNPs and cancer risk are limited. In 2012, a genome‐wide association study was carried out on Wilms tumor. The authors used cases recruited through oncology clinics in North America to identify genetic variants that confer susceptibility to Wilms tumor. They selected SNPs that demonstrated an association of a significance level of *p *< 5 × 10^−5^ for the replication phase. They failed to detect *YTHDF1* gene SNPs that were associated with Wilms tumor risk.^12^ Meng et al.^37^ genotyped 240 SNPs in 20 m^6^A modification‐related genes on colorectal cancer in China. Two SNPs rs2024768 and rs6090289 in the *YTHDF1* gene could not modify the risk of colorectal cancer. We also investigated the role of *YTHDF1* gene SNPs on the risk of hepatoblastoma using 313 hepatoblastoma cases and 1446 controls from China.^29^ For the two SNPs analyzed, rs6011668 C>T could not impact hepatoblastoma risk, but rs6090311 G allele could decrease hepatoblastoma risk. In the current study, no significant relationships were detected between rs6011668 C>T or rs6090311 A>G and Wilms tumor risk, respectively. Thus, the role of *YTHDF1* SNPs varies from types of cancers.

We admit that our study has its weakness. First, the sample size, although enrolled from several hospitals, may be too small to explain the effects of an entire population. Second, we had no access to other environmental factors, which could have biased Wilms tumor risk assessment without adequate adjustment for these covariates in the risk evaluation model. Third, all the participants were Chinese, and the applicability of the findings to other populations requires confirmation.

In conclusion, our study did not find strong evidence that *YTHDF1* gene variants influence Wilms tumor risk. Our results require independent replication in larger studies, preferably with more detailed information on environmental effect analysis, functional experiments, and across other populations.

## CONFLICT OF INTEREST

None.

## Supporting information

Table S1

## Data Availability

The data used in the current study are available from the corresponding author upon reasonable request.
