# Supplementary material for: Impact of YTHDF1 gene polymorphisms on Wilms tumor susceptibility: A five‐center case‐control study
Source: J Clin Lab Anal. 2021 Jun 21;35(8):e23875. doi: 10.1002/jcla.23875 (PMC8373325; doi:10.1002/jcla.23875)
Supplement: Supplementary file 1 — Table S1 [file JCLA-35-e23875-s001.doc]

| **Table S1**. Frequency distribution of selected variables in Wilms tumor patients and cancer-free controls | | | | | |
| --- | --- | --- | --- | --- | --- |
| Variables | Cases (N=414) | | Controls (N=1199) | | *P* ^a^ |
|  | No. | % | No. | % |  |
| Age range, month | 1-148.63 | | 0.03-156 | | 0.118 |
| Mean ± SD | 31.14 ± 24.27 | | 32.31 ± 26.15 | |  |
| ≤18 | 143 | 34.54 | 466 | 38.87 |  |
| >18 | 271 | 65.46 | 733 | 61.13 |  |
| Sex |  |  |  |  | 0.218 |
| Female | 194 | 46.86 | 520 | 43.37 |  |
| Male | 220 | 53.14 | 679 | 56.63 |  |
| Clinical stages |  |  |  |  |  |
| I | 137 | 33.09 | / | / |  |
| II | 116 | 28.02 | / | / |  |
| III | 94 | 22.71 | / | / |  |
| IV | 49 | 11.84 | / | / |  |
| NA | 18 | 4.35 | / | / |  |
| SD, standard deviation; NA, not available.  ^a^ Two-sided χ^2^ test for distributions between Wilms tumor patients and cancer-free controls. | | | | | |
